# Is Dietitian Use Associated with Celiac Disease Outcomes?

**DOI:** 10.3390/nu5051585

**Published:** 2013-05-15

**Authors:** SriHari Mahadev, Suzanne Simpson, Benjamin Lebwohl, Suzanne K. Lewis, Christina A. Tennyson, Peter H. R. Green

**Affiliations:** Celiac Disease Center, Columbia University College of Physicians and Surgeons, 180 Fort Washington Avenue, Suite 934, New York, NY 10032, USA; E-Mails: sm3455@columbia.edu (S.M.); sms2246@columbia.edu (S.S.); bl114@columbia.edu (B.L.); skl3@columbia.edu (S.K.L.); ct2398@columbia.edu (C.A.T.)

**Keywords:** celiac disease, dietary services, quality of life, quality improvement

## Abstract

A gluten-free diet (GFD) is the treatment for celiac disease (CD), but due to its complexity, dietitian referral is uniformly recommended. We surveyed patients with CD to determine if dietitian use is associated with quality of life, symptom severity, or GFD adherence. The survey utilized three validated CD-specific instruments: the CD quality of life (CD-QOL), CD symptom index (CSI) and CD adherence test (CDAT). Four hundred and thirteen patients with biopsy-proven CD were eligible for inclusion. The majority (77%) were female and mean BMI was 24.1. Over three-quarters of patients (326, 79%) had seen a dietitian, however, 161 (39%) had seen a dietitian only once. Age, sex, and education level were not associated with dietitian use; nor was BMI (24.6 *vs*. 24.0, *p* = 0.45). On multivariate analysis, adjusting for age gender, education, duration of disease, and body mass index, dietitian use was not associated with CD-QOL, CSI, or CDAT scores. Our survey did not show an association between dietitian use and symptom severity, adherence, or quality of life. Delay in diagnosis was associated with poorer outcomes. This is a preliminary study with several limitations, and further prospective analysis is needed to evaluate the benefits and cost-effectiveness of dietitian-referral in the care of celiac disease patients.

## 1. Introduction

Celiac disease (CD) is a common multi-system autoimmune disease, affecting approximately 1% of people worldwide [1]. Predisposed individuals develop an immune response to gluten, a protein found in the cereal grains: wheat, barley and rye. Autoimmune intestinal damage is the cardinal feature of CD, and typically involves villous atrophy, crypt hyperplasia, and increased intraepithelial lymphocytes [2]. Symptoms may be subclinical, varying from gastrointestinal upset to severe malabsorption [3,4]. Skin, nervous system, and multisystem involvement is also recognized. Strict avoidance of gluten-containing foods can reverse both enteric and extra-intestinal manifestations of the disease. 

CD is unique in that its treatment consists of a dietary intervention: lifelong exclusion of gluten. A gluten-free diet (GFD) is highly effective at improving symptoms of CD in the majority of patients [5]. Nevertheless, a significant proportion remains symptomatic, and lack of strict adherence to GFD is the primary cause [6]. Those patients with celiac disease who follow a GFD frequently have persistent villous atrophy which may result from persistent gluten contamination and, in rare cases, can predispose patients to serious sequelae including T-cell lymphoma [7]. 

Although straightforward in principle, strict avoidance of gluten is challenging in practice. Gluten-containing products are ubiquitous and contamination may occur both consciously and unintentionally [8]. Poor labeling can make it difficult to determine which foods are gluten free, and options may be limited when eating out and traveling [9]. Moreover, a GFD is significantly more expensive, and may be deficient in certain nutrients, when compared to a regular diet [10,11]. Given the complexity of maintaining a strict GFD, multiple guidelines recommend dietetic referral for patients diagnosed with CD [12,13,14,15]. Dietitian involvement was recommended in the National Institutes of Health (NIH) Consensus Development Conference on celiac disease (2004) [16]. There is evidence that when asked to choose among several referral options, patients themselves express a preference for dietetic follow-up [17]. Nevertheless, availability of expert dietetic counselors is limited and may impact upon patient outcomes [18]. Membership in celiac advocacy groups, and regular dietetic follow-up has previously been reported to be correlate with higher rates of GFD adherence [19,20]. However there have been no studies that have directly examined the impact of dietitian use on celiac disease outcomes in the United States. As such, we sought to determine if dietitian use was associated with quality of life, symptom severity, or GFD adherence in patients with celiac disease.

## 2. Methods

Approval was obtained from the Institutional Review Board of Columbia University Medical Center prior to initiation of the study.

Adults (≥18 years of age) with celiac disease were recruited to participate in a survey either via email or in-person. A link to an online questionnaire, hosted by SurveyMonkey, was distributed by the Celiac Disease Center of Columbia University to an email list of patients affiliated with the Center. In addition, the questionnaire was administered to attendees at celiac support group conferences in Iowa, California, and New York, and patients additionally completed the questionnaire on-paper during an office visit. Data was collected between November 2010 and July 2011. Prior to distribution, the questionnaire was administered to a group of ten patients and subsequently modified for clarity. 

The survey consisted of questions on demographics, celiac disease onset, symptoms, and dietitian use. It also included three validated celiac disease-specific instruments to assess quality of life, disease activity, and GFD adherence respectively [21,22,23]. Patients were asked how many times they had seen a dietitian: never, once, or more than once. Patients were excluded from the analysis if they did not have biopsy-proven celiac disease, or omitted the items on gender, age, or dietitian use. 

The celiac disease-specific quality of life instrument (CD-QOL) was used to assess quality of life [21]. This validated instrument consists of 20 questions across four clinically relevant subscales (celiac disease-related limitations, dysphoria, health concerns, and inadequate treatment), and asks the respondent to indicate the frequency of celiac disease-related symptoms over the previous 30 days. The questions are graded on a 5-point Likert scale labeled 1 through 5, where 1 = not at all, 2 = slightly, 3 = moderately, 4 = quite a bit, and 5 = a great deal. The responses were reverse-coded and summed, with a higher score (up to a maximum of 100) suggestive of higher quality of life. No clear cut-off point has been established to dichotomize CD-QOL scores; hence membership in the lowest quartile of CD-QOL was taken to indicate poorer quality of life. 

The Celiac Symptom Index (CSI) was employed to assess celiac disease-specific symptom severity [23]. The CSI consists of 16 questions on a 5-point Likert scale; with scores ≤30 and ≥45 suggestive of clinical remission and ongoing active disease respectively. For the purpose of our analysis; a single cutoff of ≥35 (suggestive of ongoing disease) was used to dichotomize patients. 

The Celiac Disease Dietary Adherence Test (CDAT) was used to assess adherence to a GFD [22]. This validated 7-question instrument employs a 5-point Likert scale, with additive scores ranging from 7 to 35, where higher scores indicate worse adherence. For the purposes of dichotomization, scores ≥13 were taken to be indicative of poor adherence. 

Univariate analysis was used to identify associations between demographics, dietitian use, and CD-QOL, CSI, and CDAT. The Chi-square and Fisher exact tests were used to compare proportions of categorical variables. The Mann-Whitney U test was used to compare continuous variables. Logistic regression was performed to develop a multivariate model identifying variables predictive of three outcomes as determined by these validated scores: poor quality of life, high symptom activity, and poor adherence. Two-sided *p*-values <0.05 were considered significant. All statistical calculations were performed with SAS 9.2 (Cary, NC, USA).

## 3. Results

### 3.1. Patient Characteristics

Of 600 respondents, 413 with biopsy-proven celiac disease were eligible for inclusion (Table 1). Roughly equal numbers of patients completed the survey online (47%) *versus* on paper (49%). The majority (77%) of subjects were female, with almost one-quarter over the age of 60 (24%). The cohort was highly educated, with 88% having attained a college degree, and 40% having a graduate or higher degree. 

**Table 1:** Patient characteristics.

		*n* (%)
Total number		413
*Age*		
	18–30	80 (19)
	31–40	67 (16)
	41–50	75 (18)
	51–60	93 (23)
	61–70	67 (16)
	>70	31 (8)
*Gender*		
	Male	94 (23)
	Female	319 (77)
*Educational level*		
	High school or less	43 (10)
	College	197 (48)
	Graduate school	167 (40)
*Presentation*		
	Classical	166 (40)
	Atypical	204 (49)
	None	32 (8)
*Years since diagnosis*		
	<1	55 (13)
	1–4	149 (36)
	5–10	115 (28)
	>10	93 (23)
*Delay to diagnosis in years*		
	<1	51 (12)
	1–4	149 (36)
	5–10	115 (28)
	>10	93 (23)
Mean Body Mass Index		24.1
*Symptoms improved on GFD*		
	Yes	288 (70)
	Somewhat	73 (18)
	No	28 (7)
*Filled in survey*		
	Online	195 (47)
	Paper	204 (49)

### 3.2. Disease Characteristics

Regarding CD presentation, almost half of patients (49%) reported atypical symptoms of fatigue, anemia or osteoporosis; 40% reported classical diarrhea-predominant symptoms, and 8% reported no symptoms. Most patients’ symptoms were either improved (70%) or somewhat improved (13%) with GFD. Mean body mass index (BMI) was in the normal-weight range (24.1). The median time since diagnosis was 5–10 years, and the median delay from onset of symptoms to diagnosis was also 5–10 years. 

### 3.3. Dietitian Use

Of the 413 patients in the analysis, 326 (79%) reported having seen a dietitian, but 161 (39%) had only seen a dietitian once. One hundred and sixty-four patients (40%) agreed with the statement “it is hard to find a dietitian knowledgeable about GFD”. One hundred and ninety-one patients (46%) reported gaining weight since starting a GFD. Demographic factors, including age, sex, and education level, were not associated with dietitian use (Table 2). There was no significant difference in BMI between patients who had and had not seen a dietitian (24.0 *vs.* 25.6, *p* = 0.45). Patients who had not seen a dietitian were more likely to agree with the statement “health insurance limits my ability to see a dietitian” (55% *vs.* 43%; *p* = 0.04).

**Table 2:** Patient characteristics by dietitian use.

	Not seen *n* (%)	Seen *n* (%)	*p*
Total number	87 (100)	326 (100)	
Age ≥60	20 (23)	78 (24)	0.96
Female gender	72 (83)	247 (76)	0.21
College educated	75 (86)	289 (89)	0.53
Symptoms improved on GFD	63 (72)	225 (69)	0.54
Mean BMI	25.6	24.0	0.45

### 3.4. Quality of Life, Disease Activity, and Dietary Adherence

Dietitian use was not associated with CD-QOL, CDAT, or CSI on univariate analysis (Table 3). Multivariate analysis (Table 4) identified two covariates associated with low CD-QOL, indicative of poor quality of life: a long delay (>10 years *vs.* <1 year) from symptom onset to CD diagnosis (OR 3.92, 95% CI 1.45–0.63), and underweight *vs*. normal weight (OR 3.46, 1.12–10.68). Older age (>60) and time since diagnosis (>10 years *vs.* <1 year) were protective for disease activity as measured by CSI (OR 0.35, 95% CI 0.17–0.71 and OR 0.34, 95% CI 0.13–0.87 respectively). Dietitian use was not associated with CD-QOL, CSI, or CDAT scores on multivariate analysis (see Table 4).

**Table 3:** Mean validated scores and use of a dietitian.

	Not seen	Seen	*p*
CD-QOL	75.4	72.6	0.08
CSI	33.5	33.3	0.62
CDAT	12.9	12.1	0.11

**Table 4:** Multivariate analysis of factors associated with low celiac disease quality of life (CD-QOL), high CD symptom index (CSI), and high CD adherence test (CDAT).

Covariate	Low CD-QOL		High CSI		High CDAT	
	OR	95% CI	OR	95% CI	OR	95% CI
Seen a dietitian	1.49	0.68–3.24	0.86	0.45–1.63	0.85	0.47–1.55
Older age (>60)	0.60	0.26–1.37	0.35	0.17–0.71	0.53	0.28–1.01
Male gender	1.29	0.61–2.72	1.24	0.58–2.64	0.92	0.48–1.77
College educated	3.65	0.97–13.7	1.33	0.53–3.38	0.74	0.32–1.71
Time since diagnosis ^1^	0.38	0.13–1.10	0.34	0.13–0.87	0.48	0.20–1.13
Delayed diagnosis ^1^	3.92	1.45–10.63	2.08	0.98–4.41	1.05	0.53–2.07
Underweight *vs.* normal	3.46	1.12–10.68	2.52	0.79–8.04	1.90	0.66–5.51
Overweight *vs.* normal	1.31	0.62–2.75	0.60	0.31–1.16	0.66	0.36–1.21
Obese *vs.* normal	0.86	0.33–2.23	0.75	0.32–1.76	0.65	0.29–1.46

^1^ >10 years *vs.* <1 year.

## 4. Discussion

To our knowledge, this is the first study to examine dietitian use and outcomes as measured by validated CD-specific instruments. We were surprised at the lack of association between dietitian use, dietary adherence and quality of life. The study has several limitations, including selection bias, self-reported outcomes, and poor generalizability, however prior literature addressing these issues is similarly limited. While our findings are preliminary, they should provide impetus to further study the role of dietary counseling in CD management.

Several studies have examined dietitian use in CD, with frequently divergent results. Ukkola *et al.* [24] surveyed 698 newly diagnosed CD patients on their perceptions of living with CD, and found no correlation between dietitian follow-up and patients’ knowledge of GFD or experience of their disease. A similar proportion of patients in this cohort (76%) had received dietitian counseling as in our sample (79%). The authors note that despite a lack of correlation, patients often requested more detailed dietary counseling when asked to indicate in their own words their wishes/needs, suggesting insufficient dietitian contact. 

One outcome measure that has been demonstrated to be associated with dietitian use is improved GFD adherence, but this was not seen in our cohort. Wylie *et al*. [25] concluded in a prospective cohort of 99 patients that annual review within the context of a dietitian-led celiac clinic can significantly improve adherence as well as other nutritional markers. In a systemic review of 38 studies examining factors associated with adherence to GFD, Hall *et al*. [19] also concluded that regular dietetic follow-up and annual review in a dietitian-led clinic can improve adherence. Membership of a patient support group has been associated with adherence, and studies with cohorts recruited from patient support groups trend towards higher adherence rates (66%–90% strict adherence) than clinical samples (42%–91%) [26]. There are several possible reasons why these results might diverge from that of our study. First, dietitian use was common in our study (approaching 80%), leading to a small sample size for patients who had not seen a dietitian, and hence decreased ability to detect a difference. Our cohort was very highly educated, with almost 90% of patients having a college degree or higher qualification, as compared to a US-wide average of 28% [27]. It may be that university-educated patients may have less to gain from dietitian referral: they are more health-literate at baseline, and are better equipped to seek out and utilize information from other sources in addition to their health professional. As such, the high prevalence of highly educated patients in our cohort may have reduced the effect size of dietitian exposure. Hall *et al.* [19] included studies that assessed GFD adherence via a trained nutritionist assessment, which may be more sensitive for differences than use of the self-reported CDAT questionnaire. Last, cultural factors can influence response to dietary interventions. In a cross-sectional survey from 2004, Butterworth *et al*. [20] noted that South Asian patients with CD were much less likely than Caucasians to be members of a support group (53% *vs.* 80%, *p* = 0.02), were more frequently dissatisfied with dietetic advice (30% *vs.* 6%, *p* = 0.01), and exhibited a trend towards poorer dietetic follow-up (31% *vs.* 60%, *p* = NS). Dietetic follow-up correlated with GFD compliance for Caucasians but not for South Asians in that study. It is unclear to what extent these cultural issues apply to our cohort.

Although dietitian use was common in this study, almost half of these patients had only seen a dietitian once, possibly at diagnosis, falling short of CD treatment guidelines. Guidelines published by several authorities generally recommend annual dietitian review. The American Gastroenterological Association position statement on diagnosis and management of CD advises consultation with an experienced dietitian at initiation of a treatment plan and ongoing evaluation at regular intervals by a health care team including a dietitian [12]. Several other organizations also publish guidelines recommending ongoing dietitian involvement, including the NIH, the United States Department of Health and Human Services Agency for Healthcare Research and Quality, the National Institute for Health and Clinical Excellence in the United Kingdom, and the World Gastroenterology Association [14,16,28,29]. Given a median time since diagnosis of 5–10 years in this cohort, dietitian involvement fell well short of recommendations in a significant proportion of patients and insufficient dietitian follow-up may have contributed to the lack of effect on outcomes.

A long delay of >10 years from symptom onset to diagnosis of CD was associated with poor quality of life in this cohort. Prior studies have addressed the influence of delayed diagnosis on CD outcomes. In a survey of over 1000 patients with CD from Sweden, the mean delay to diagnosis from first symptoms was 9.7 years, and 5.8 years from the first doctor visit [30]. A long delay was associated with lower quality-adjusted life year scores prior to treatment; however delay in diagnosis had no effect on scores following initiation of GFD. The study concluded that untreated CD resulted in poor quality of life, which returned to baseline with treatment. Our findings of persistent quality of life impairment even following initiation of GFD are novel and warrant further study. 

The limitations of this study include recruitment from a tertiary referral center (the Celiac Disease Center of Columbia University) and support groups, leading to a cohort that is likely to have greater health literacy than average. The sample size for patients who had not seen a dietitian was low, limiting our power to detect a difference in CD outcomes. The actual response rate of the survey is unable to be determined as the survey link may have been electronically forwarded by subjects to other members of support groups or known contacts with celiac disease. In addition we did not have information on the quality, nor expertise and practice setting of the dietitians used by the respondents.

## 5. Conclusions

In this survey of patients with celiac disease, more than 20% of respondents had never seen a dietitian, and 39% only saw a dietitian once. Dietitian follow-up fell short of published guidelines, which may relate to insurance access issues. Dietitian exposure was not associated with symptom severity, adherence, or quality of life, while delay in diagnosis was associated with poorer quality of life. Further prospective analysis is needed to evaluate the benefits and cost-effectiveness of dietitian referral in the care of patients with celiac disease.
